# Clinical Application of a Neurosurgical Robot in Intracranial Ommaya Reservoir Implantation

**DOI:** 10.3389/fnbot.2021.638633

**Published:** 2021-03-26

**Authors:** Huan-Guang Liu, De-Feng Liu, Kai Zhang, Fan-Gang Meng, An-Chao Yang, Jian-Guo Zhang

**Affiliations:** ^1^Department of Neurosurgery, Beijing Tiantan Hospital, Capital Medical University, Beijing, China; ^2^Department of Functional Neurosurgery, Beijing Neurosurgical Institute, Capital Medical University, Beijing, China; ^3^Beijing Key Laboratory of Neurostimulation, Beijing, China

**Keywords:** Ommaya reservoir implantation, robot-assisted, minimally invasive, safety, efficiency

## Abstract

**Background:** The Ommaya reservoir implantation technique allows for bypass of the blood-brain barrier. It can be continuously administered locally and be used to repeatedly flush the intracranial cavity to achieve the purpose of treatment. Accurate, fast, and minimally invasive placement of the drainage tube is essential during the Ommaya reservoir implantation technique, which can be achieved with the assistance of robots.

**Methods:** We retrospectively analyzed a total of 100 patients undergoing Ommaya reservoir implantation, of which 50 were implanted using a robot, and the remaining 50 were implanted using conventional surgical methods. We then compared the data related to surgery between the two groups and calculated the accuracy of the drainage tube of the robot-assisted group.

**Results:** The average operation time of robot-assisted surgery groups was 41.17 ± 11.09 min, the bone hole diameter was 4.1 ± 0.5 mm, the intraoperative blood loss was 11.1 ± 3.08 ml, and the average hospitalization time was 3.9 ± 1.2 days. All of the Ommaya reservoirs were successful in one pass, and there were no complications such as infection or incorrect placement of the tube. In the conventional Ommaya reservoir implantation group, the average operation time was 65 ± 14.32 min, the bone hole diameter was 11.3 ± 0.3 mm, the intraoperative blood loss was 19.9 ± 3.98 ml, and the average hospitalization time was 4.1 ± 0.5 days. In the robot-assisted surgery group, the radial error was 2.14 ± 0.99 mm and the axial error was 1.69 ± 1.24 mm.

**Conclusions:** Robot-assisted stereotactic Ommaya reservoir implantation is quick, effective, and minimally invasive. The technique effectively negates the inefficiencies of craniotomy and provides a novel treatment for intracranial lesions.

## Introduction

Intracranial space-occupying lesions, especially those with large space-occupying volume and those located in important functional areas, are difficult to remove completely, and result in high recurrence and poor prognosis in patients. Therefore, prolonging the survival time of patients and improving their quality of life have become the main purpose of surgery. The Ommaya reservoir is a ventricular drainage device invented by Ommaya AK in 1963 (Ommaya, 1963). One of the main functions of the Ommaya reservoir is to enable drugs to directly bypass the blood-brain barrier for intraventricular administration, reduce intracranial hypertension, and improve the symptoms of increased intracranial pressure. Furthermore, cerebrospinal fluid can be repeatedly extracted for examination and analysis to understand the curative effect and determine the concentration of drugs in the ventricles. In recent years, the scope of clinical application of the Ommaya reservoir has gradually expanded, and there are increasing numbers of reports on the treatment of intracranial space occupying lesions. In a previous work, the process of placing the drainage tube operated under non-direct vision was investigated, with results indicating several complications, including intraoperative intracranial hemorrhage, infection and incorrect placement of the drainage tube. With the development of microsurgery and the popularization of the concept of minimally invasive treatment, neuronavigation has become a routine auxiliary technology for neurosurgeons. With the help of navigation (Wang A. et al., 2019), catheterization can be located accurately in real time, so as to avoid damage to important brain tissues and blood vessels, and improve the accuracy and safety of neurosurgery. However, the neuronavigation operation is relatively complex and expensive, and increases the duration of the operation, which limits its use in clinical practice. Robot-assisted stereotactic technology has the main characteristics of accurate positioning and less invasiveness. As an important part of neurosurgery, robot-assisted stereotactic technology is playing an increasingly important role in several procedures, including deep brain stimulation (DBS), stereoelectroencephalography (SEEG), and stereotactic brain biopsy (SBB). Importantly, the neurosurgical robot also offers the advantages of accuracy, stability, and safety, which can meet the needs of Ommaya reservoir implantation.

## Materials and Methods

### General Data

The clinical data of tumor patients who underwent robot-assisted Ommaya reservoir implantation in the Department of Neurosurgery of Beijing Tiantan Hospital affiliated to Capital Medical University from August 2016 to September 2020 were analyzed retrospectively. A total of 50 patients, including 29 males and 21 females, were included in the group of robot-assisted, and 17 of which underwent robot-assisted biopsy and Ommaya reservoir implantation. The patient age range was from 1 to 68 years old, with an average of 17.15 ± 21.71 years. As a control, we selected 50 patients who underwent conventional surgery to implant the Ommaya reservoir during the same period, including 26 males and 24 females. The age range was from 1 to 65 years old, with an average of 20.05 ± 20.52 years (Table 1). All the patients in this study provided informed consent and signed the operation informed consent form. This study was approved by the Ethics Committee of Beijing Tiantan Hospital (Grant No. QX201600-706).

**Table 1:** Patient characteristics.

**Variables**	**Robot-assisted ommaya reservoir implantation (*n* = 50)**	**Conventional ommaya reservoir implantation (*n* = 50)**	***P* value**
Age (M ± SD)	6.98 ± 4.30	8.62 ± 4.47	0.064
Gender, *N* (%)			
Male	29 (58%)	26 (52%)	
Female	21 (42%)	24 (48%)	

### Image Preprocessing

All patients underwent magnetic resonance imaging (MRI) before surgery (3.0 Tesla, Siemens, Germany). Specifically, to guarantee the visualization of the anatomical structures of interest, sagittal and axial volumetric T1-weighted images (slice thickness 1.0 mm, TR 6.4 ms, TE 3.0 ms, interslice gap 0 mm, flip angle 8°) were analyzed.

**Figure 1:**
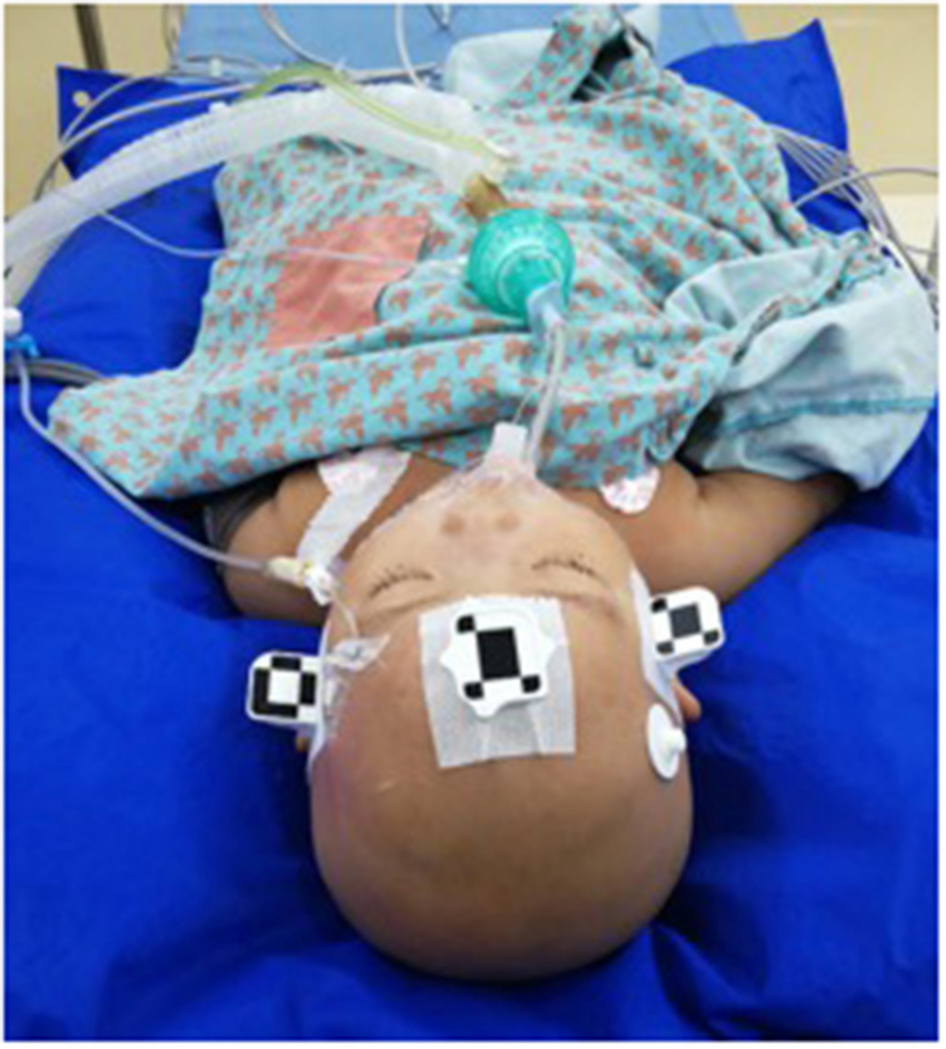
The dedicated videometric-tracked marker was fastened to the scalp.

On the day of surgery, a dedicated videometric-tracked marker referred to as the optical frame marker, which was capable of automatic patient-to-image registration, was attached to the scalp (Figure 1). The optical frame marker consisted of target patterns comprising checkered target regions referred to as ‘XPoint’ that could be pinpointed on video image sequences and fiducial balls. After, an axial volumetric computed tomography (CT; slice thickness 0.625 mm, interslice gap 0 mm, 120 kVp) scan was taken. All images were loaded into Remebot software, and T1-weighted images were overlayed with the CT images and used as the reference due to MRI distortions.

### Procedures

Together two experienced neurosurgeons identified the target lesion before surgery.

**Figure 2:**
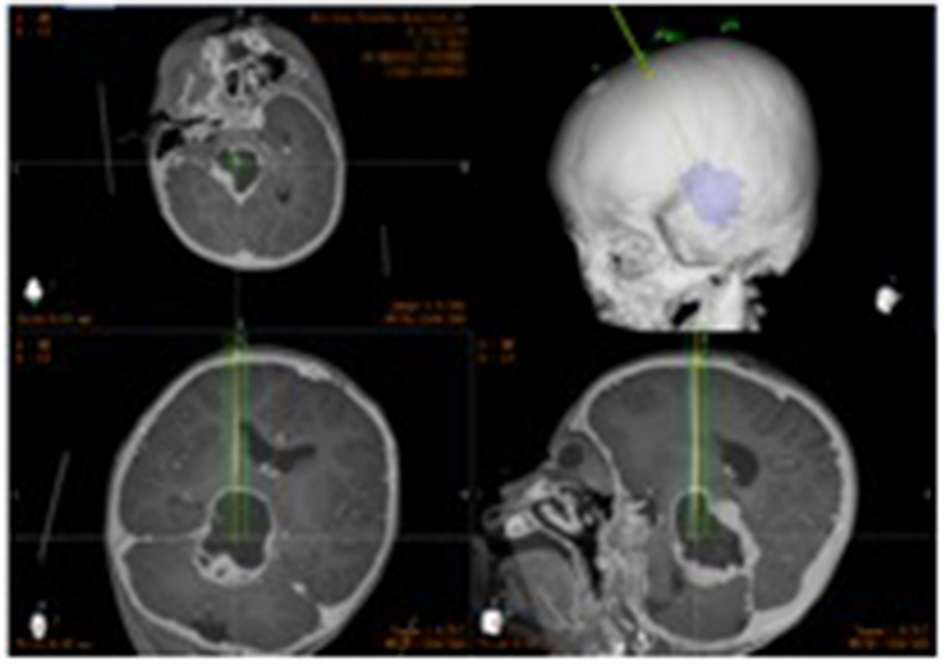
The target and cranial path before surgery.

After segmenting the 3D objects of interest, surgical planning was performed. The trajectory required to reach that location was planned on 3D objects or any available view, avoiding vessels and nerves (Figure 2).

**Figure 3:**
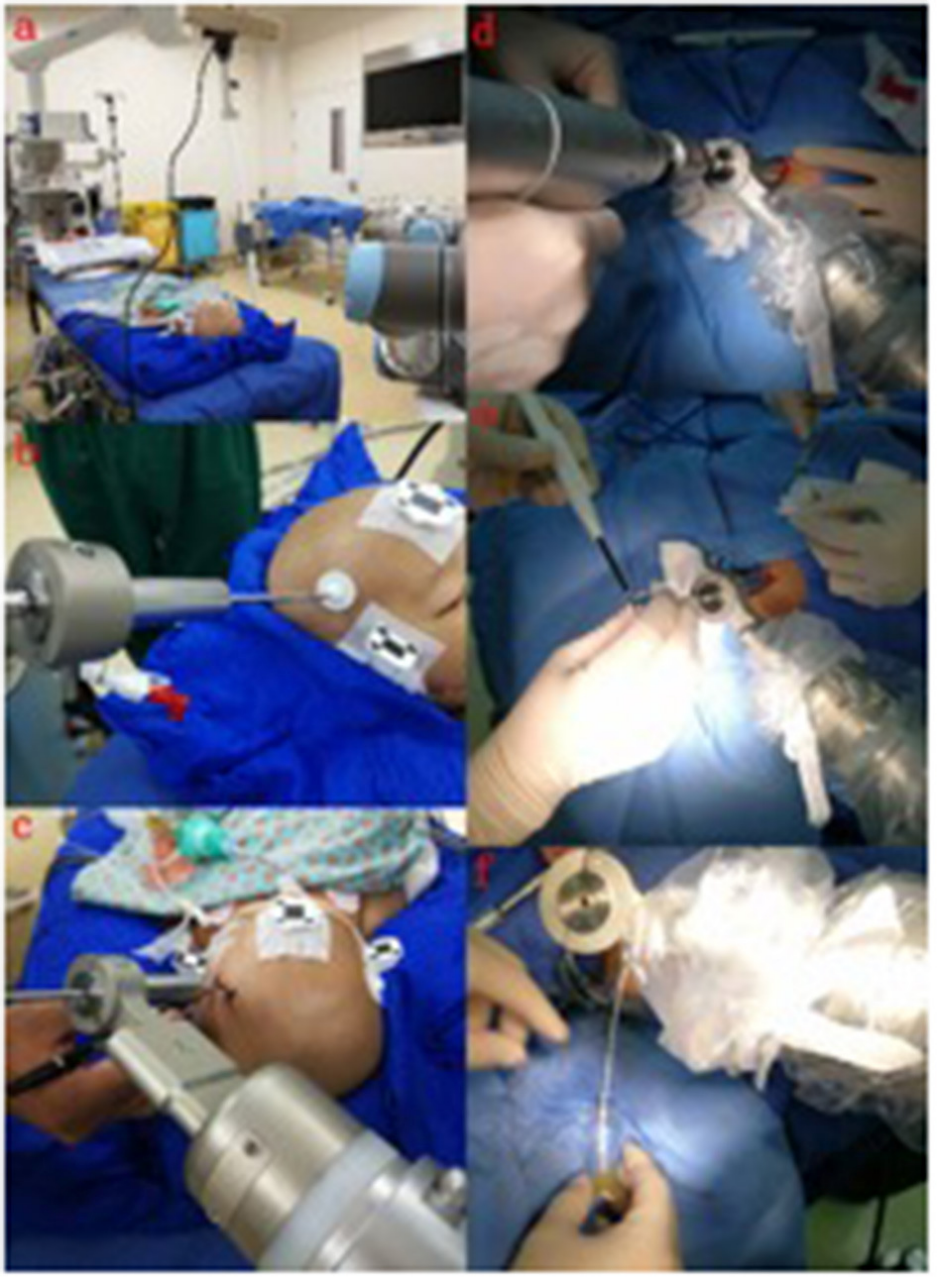
The process of implantation. **(a)** The optical frame marker was detected; **(b)** the arm repositioned itself; **(c)** the scalp entry points were marked; **(d)** burr-hole drilling was performed under the guidance of the robotic arm; **(e)** electrocoagulation punctures in the dura mater; **(f)** fluid can be seen flowing out after the drainage tube was implanted in the target area.

In the operating room, the patient was placed in the supine position, and the head was fixed in the sponge padding head pillow. A videometric tracker (MicronTracker, ClaroNav, Canada), with three stereotactic cameras held by an independent stand, was installed above the patient's head where the optical frame marker could be detected within the tracker's field of measurement (Figure 3a). Next, correlations of the different spaces were carried out, involving two steps, namely: (1) tracker-to-image registration and (2) tracker-to-robot registration. The Remebot robotic system features a paired point-based, automatic registration. On any view of the preoperative CT images, one fiducial point was automatically marked by calculating the center of a series of high-contrast circular zones. At the end of the tracker-to-image registration, the registration error was validated if the space was less than 0.3 mm. The tracker-to-robot registration was achieved by correlating two sets of spatial positions from the robotic arm space and the tracker space. A fiducial point was defined on the videometric-tracked target pattern engraved on the end effector attached to the robotic arm (Figure 3b). During registration, the robotic arm automatically moved to certain areas surrounding the patient's head, and the coordinates of that fiducial point in the separate spaces were automatically obtained from the robot forward calculation and the tracker. At the end of the tracker-to-robot registration, the registration error was validated if the space was < 0.08 mm. Subsequently, the robot-to-image registration was accomplished by relying on the above correlations, and data could be transferred between the images and the robotic arm.

Following the registration, the robotic arm was oriented on command to the bilateral trajectories, and the scalp entry points were marked (Figure 3c). After draping and local anesthesia, scalp incisions and burr-hole drilling were performed under the guidance of the robotic arm, but the dura was preserved to prevent untimely cerebral spinal fluid loss and subsequent brain shift (Figure 3d). In case of any possible displacement of the patient's head, the automatic registration was efficiently repeated. Once completed, registration was visually inspected and an accuracy of 0.5 mm was guaranteed by commanding the robotic arm to guide a tooltip, 1 mm in diameter into two holes, each 2 mm in diameter, on the optical frame marker, according to the preoperative planning. After, the robotic arm moved to a target point and was oriented to the trajectory with a micro-drive device. The dura was perforated (Figure 3e), and cannulas were advanced to the defined depth.

The drainage tube entered the target along the cannula, and the patient's vital signs and symptoms were observed throughout the process. When necessary, the drainage tube was altered through micro-movements of the robotic arm by sub-millimeter steps as small as 0.1 mm. The drainage tube was then implanted in the target area and the fluid was observed (Figure 3f). When the physiological and clinical criteria for successful tube placement were fulfilled, the drainage tube was anchored to the skull. After the successful catheterization, the drainage tube was connected to the Ommaya capsule and fixed under the scalp within 2 cm of the burr hole.

All patients had a postoperative CT scan (slice thickness 0.625 mm, interslice gap 0 mm, 120 kVp). The CT images were matched with the preoperative planning to assess the accuracy of the drainage tube placement.

The accuracy of the drainage tube was defined as the deviation between the actual center of the implanted tube and the intended target point, and was assessed using two types of measurements: the “radial error (RE),” defined as the scalar distance measured from the view perpendicular to the planned trajectory; and the “axial error (AE),” defined as the scalar distance along the planned trajectory measured from the view along the planned trajectory. All patients were followed up to verify associated complications, such as hemorrhage, infection, or poor incision healing.

### Conventional Ommaya Reservoir Implantation

The frontal angle of the lateral ventricle was selected as the puncture direction, as the frontal angle of the right lateral ventricle is usually selected as the puncture target. A straight intrahair incision was made, a single hole was drilled in the skull, and the dura was cut with a sharp knife. The ventricular end of the Ommaya reservoir catheter was punctured through the skull and into the frontal horn of the lateral ventricle to enable smooth drainage of the cerebrospinal fluid. The catheter was connected to the reservoir, and the reservoir was buried under the scalp 1 to 2 cm from the incision. Finally, the wound was sutured.

### Statistical Analysis

SPSS 23.0 software (IBM SPSS Statistics Inc., Chicago, IL, USA) was used for statistical analysis. The experimental measurement results are expressed as mean ± standard deviation (x ± s). The normality and homoschedasticity of the two groups of data were detected. If the variance is aligned, one-way analysis of variance is performed, and if the variance was not uniform, the Wilcoxon tests is performed. *P* < 0.05 was considered statistically significant.

## Results

### Surgery

**Figure 4:**
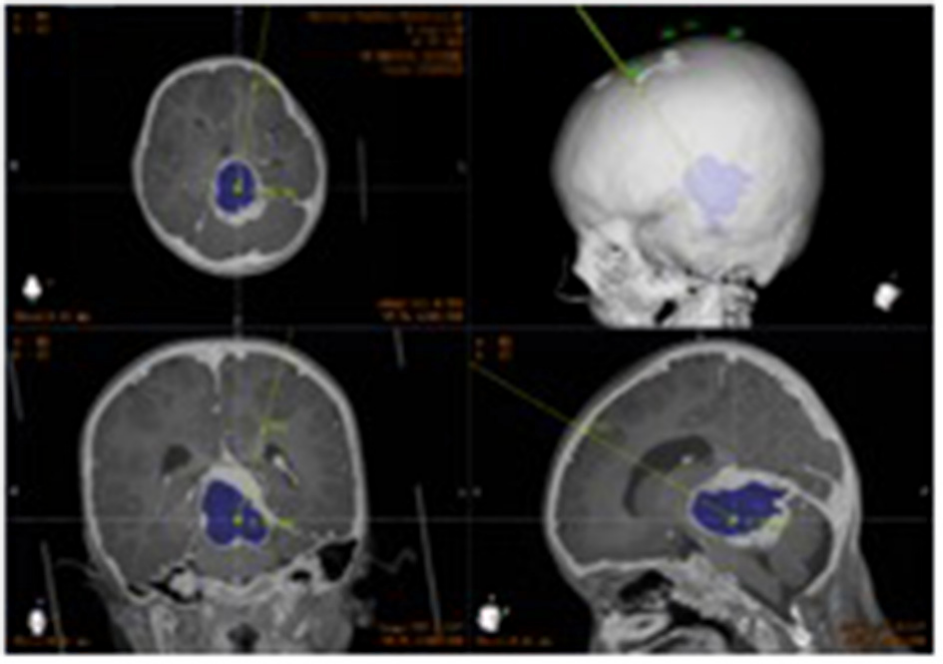
Postoperative CT. Postoperative image fusion, 3D reconstruction of the position of the intracranial drainage tube.

Ommaya reservoir implantation was successfully performed in 50 patients, including robot-assisted stereotactic biopsy and Ommaya reservoir implantation in 17 patients. There were no operation-related complications such as intracranial hemorrhage, local scalp blood supply disorder, skin and soft tissue infection, or incorrect placement of the tube (Figure 4).

### Comparison of Clinical Characteristics in the Robot-Assisted and Conventional Surgery Groups

The average operation time (from the time the patient enters the operating room to the end of the operation) of the 50 patients was 41.17 ± 11.09 min, the bone hole diameter was 4.1 ± 0.5 mm, the intraoperative blood loss was 11.1 ± 3.08 ml, and the average hospitalization time was 3.9 ± 1.2 days (Table 2). All of the Ommaya reservoirs were successfully implanted during the first attempt, and there were no complications such as infection or incorrect placement of the tube. Two patients experienced a small amount of bleeding at the puncture site after the surgery; however, no interventional treatment was required. Among the patients, 17 underwent robot-assisted stereotactic biopsy while implanting the Ommaya reservoir.

**Table 2:** Comparison of clinical characteristics in the robot-assisted and conventional surgery groups.

**Variables**	**Robot-assisted ommaya reservoir implantation (*n* = 50)**	**Conventional ommaya reservoir implantation (*n* = 50)**	***p* Value**
Implant position			
Ventricle	10	50	
The lumen of the tumor	40	-	
Bone hole diameter	4.1 ± 0.5 mm	11.3 ± 0.3 mm	<0.05
Operation duration	41.17 ± 11.09 min	65 ± 14.32 min	<0.01
Intraoperatve blood loss	11.1 ± 3.08 ml	19.9 ± 3.98 ml	<0.01
Successful rate of once puncture	100% (50/50)	82% (41/50)	-
The hospitalization time	3.9 ± 1.2 days	4.1 ± 0.5 days	>0.05
Surgery-related complications			-
Hemorrhage	2	10	
Infection	0	3	

In the conventional Ommaya reservoir implantation group, the operation time was 65 ± 14.32 min, the bone hole diameter was 11.3 ± 0.3 mm, and the intraoperative blood loss was 19.9 ± 3.98 ml. The above data are statistically different from the robot-assisted implantation group (Table 2).

### Accuracy of Intracranial Ommaya Reservoir Implantation in the Robot-Assisted Group

A total of 50 Ommaya reservoir were implanted in 50 patients. The RE was 2.14 ± 0.99 mm and the AE was 1.69 ± 1.24 mm. For Ommaya reservoirs implanted into the tumor cyst, the RE was 1.78 ± 0.96 mm, and for those implanted into the ventricle, the RE was 1.96 ± 0.88 mm.

## Discussion

The neuromorphic technologies will be the next stage of the development of high-performance computing, which can greatly improve the ability of data processing and machine learning. The neuromorphic technologies can better process and deal with image signals, and then affect mechanical action. A key aspect of neuromorphic technologies is to combine learning and development to adapt to local change (plasticity) and to promote evolutionary change. The emergence of neurosurgical robot is the primary application of neuromorphic technologies.

The Remebot robotic system is a frameless stereotactic product and neurosurgery assistance tool. The robot includes a computer software system, six-axis robotic arms, and camera. The surgeon can use the computer software system to observe the multi-modal images of head and plan the best surgical puncture path. The arm can aid the surgeon to accurately locate the puncture site of the operation, and it can also act as a multi-functional operation platform. Flexible multi-axis manipulators can provide tactile feedback, that is, advanced visualization functions. The camera can perform spatial mapping and real-time tracking to ensure that the robotic arm moves along the planned path to the preoperative planned position. The videometric tracker integrated by the Remebot robotic system is a commercially available third-generation stereoscopic optical tracking product. The product is fully passive and uses available visible light illumination to detect and track objects of interest, much as humans do, by triangulating 3D poses between two video cameras with overlapping projections (Choi et al., 2019). When the path needs to be modified during the operation, the robot working platform automatically registers and identifies the fixed patients, and the navigation function enables them to move to the specified position at a specific posture angle, thus realizing a more convenient and rapid registration.

Robotic neurosurgery is based on CT and MRI scanning to generate 3D coordinates, establish the mapping relationship between computer images and actual images through positioning marks, plan and virtualize brain stereotactic surgery, and finally realize the multi-sensor intelligent robotic arm assist positioning and navigation, so as to complete the operation smoothly. These attributes provide for a safer and more reliable and accurate technique compared with traditional operations. To date, neurosurgery robots have mainly been in involved in DBS, SEEG, and SBB; stereotactic needle aspiration from hematoma, cysts, and abscesses; brachytherapy; and thermal ablation of brain tumors and seizure-generating regions (De Benedictis et al., 2017; MacDonell et al., 2018; Minchev et al., 2019; Wang Y. et al., 2019; Chaitanya et al., 2020; Moran et al., 2020). This novel robot-assisted operative technique results in millimeter accuracy in drainage tube placement.

Ommaya reservoirs are used for administration of local drug delivery and cerebrospinal fluid drainage. According to reports, the most commonly used free-hand Ommaya tube implantation technique has a catheter misalignment rate of 12–40% (Kulkarni et al., 1999; Huyette et al., 2008; Saladino et al., 2009; Wilson et al., 2013). Ventricular catheter placement for these purposes requires a high degree of accuracy and security. Complications related to frame-based stereotactic catheter placement include bleeding, infection, and revision (Kennedy et al., 2015). Deviations in the position of the implanted drainage tube may cause serious complications for the patient, and even require a second operation (Peyrl et al., 2014; Pardo-Moreno et al., 2015). The importance of accurate Ommaya reservoir implantation is self-evident. In the early application, Sandberg et al. (2000) used pre-operative pneumoencephalography for ventricular dilation and intraoperative fluoroscopic guidance in 77 patients to confirm the catheter tip position. However, the incidence of complications related to Ommaya reservoir placement using this technique is as high as 9.3%, including infections, catheter malpositions, and intracranial hemorrhages. Greenfield and Schwartz (2008) used frameless navigation technology to implant Ommaya reservoirs and found that 90% were located within 5 mm of the intended target. The average operation time was 47 min, and there were no intraoperative complications. Due to excessive deviation and excessive operating times, the practicability of the navigation needs to be improved.

The implantation of Ommaya reservoirs requires sufficient accuracy, stability, and safety, all of which are possible using neurosurgical robots.

The clinical data of tumor patients who underwent robot-assisted Ommaya reservoir implantation in the Department of Neurosurgery of Beijing Tiantan Hospital affiliated to Capital Medical University from August 2016 to September 2020 were analyzed. In this study, the age range of the 50 patients included was 1–15 years, and none had serious surgery-related complications. All drainage tubes were implanted at one time, and there was no need to adjust the position of the drainage tube during or after surgery. The average operation time of 50 patients, 17 of which underwent concurrent stereotactic biopsy, was 41.17 ± 11.09 min, the intraoperative blood loss was 11.1 ± 3.08 ml, and the average hospitalization time was 3.9 ± 1.2 days. In the conventional Ommaya reservoir implantation group, where the procedure was performed free-hand, the average bone hole diameter was 11.3 ± 0.3 mm, the average operation time was 65 ± 14.32 min, and the intraoperative blood loss was 19.9 ± 3.98 ml. For the patients in the robot-assisted group, the patients were scanned by CT after pasting marker on the scalp on the day of the operation. After entering the operating room, the images need to be imported into the robot workstation for positioning operation, and then proceed to the next step. In the conventional group, the patients were located on the body surface directly according to the data of body surface anatomy and preoperative images. In order to compare the operation time between the two groups as much as possible, we calculated the time from the time the patient entered the operating room to the end of the operation. It should be pointed out that because some of the patients in the robot-assisted group needed to have biopsies performed to determine the space-occupying nature of intracranial lesions, the operation time mentioned above includes both the drainage tube implantation time and the biopsy time. To some extent, this is different from the operation time reported in other literature. However, in terms of the changing trend of operation time, the robot-assisted group was significantly less than that of the conventional operation group. The DGR-I drill (ACRA-CUT, America), used in the conventional operation, had a diameter of 11 mm, whereas the specialized bit, used in the robot stereotactic guided assisted operation, had a diameter of only 4 mm, which could directly drill through the scalp and skull; as a result, the orientation was consistent with the preoperative planning direction. The small diameter and precision of the specialized bit not only reduced the trauma, but also effectively reduced damage to brain tissue and the recovery time of patients.

By the way, a combination of stereotactic biopsy and ommaya implantation is one of advantage of our procedure, The diagnostic rate of our biopsy in 17 patients was 100%, including opticpathwayglioma, germinoma, and recurrent glioma. The outcome of 17 patients was chemoradiotherapy after defining the pathological type.

Importantly, the application of robotic tools negates the limitation of traditional implant surgery that can only be implanted into the ventricle. The Ommaya capsule is easily and conveniently implanted into the tumor cyst, and subsequent treatment can be carried out directly and quickly. In patients requiring Ommaya capsule implantation into the ventricle, robotic tools also have advantages. The robotic system can automatically calculate the patient's 3D ventricle segmentation, that is, by selecting the appropriate ventricular region threshold, the ventricular region and other tissues can be distinguished by the gray level of the image, and the pixel level can be divided to generate segments. Then the area, shape and other characteristics of the connected segments are used to distinguish and exclude non-ventricular parts, which can achieve high-precision ventricle segmentation results. In our research, the RE of the Ommaya capsule implanted in the ventricle was 2.14 ± 0.99 mm, which helped to avoid the cerebral plexus and important blood vessels during the implantation process.

In general, the results of this study indicate that the robot-assisted drainage tube implantation results in reduced intracranial injury, speedier recovery, less patient pain, increased accuracy, and higher surgical efficiency in patients compared with conventional implantation. The robotic assisted method is also suitable for young children, as the robotic system can be fixed with a professional headrest.

In our experience, for patients with small ventricles or tumor cysts, the conventional Ommaya reservoir implantation has a higher intraoperative risk, and is more likely to cause complications such as incorrect placement of drainage tube position or multiple punctures. However, when performing the robotic assisted implantation, the size of the ventricle or tumor cyst does not affect the accuracy of implantation because the drainage tube is implanted during the first attempt and is associated with a very small RE, which limits damage to neurovascular structures and the choroid plexus.

Compared with the free-hand Ommaya tube implantation technique, the robotic system has the following advantages: (a) Stable and accurate implants. Errors associated with the traditional free-hand Ommaya tube implantation technique are due to direct human error, whereas any robotic system error is a result of incorrect data inputted into the system, and not from undesirable hand movements; (b) Efficient and accurate changes to the path and target. If the entry point and/or target are altered, the robotic arm automatically moves toward the desired position; and (c) Prediction of intraoperative deviation. The deviation of the Ommaya tube implantation can be preevaluated through the marker verification point to provide a more accurate implantation.

Compared with imaging-guided Ommaya reservoir implantation, the robotic system has the following advantages: (a) A relatively shorter learning curve. With the use of robot navigation, the target and cranial entry point are set after importing data on the operating platform, and the robotic arm can then automatically perform the operation. With image navigation, the surgeon needs to spend more time learning to use relevant equipment and software; (b) Fast registration. The robotic arm in this study is based on visual positioning registration, and its registration process can be automated, which takes less time than the imaging-guided Ommaya reservoir implantation. If necessary, secondary registration can be performed quickly during the operation.

This study has some limitations. First, the number of patients that underwent robot-assisted Ommaya tube implantation was small, and the sample size needs to be expanded in the future. Second, this study was not a randomized trial, and subsequent patients should be randomly assigned to undergo both the robot-assisted and free-hand Ommaya reservoir implantation techniques.

## Conclusions

Robot-assisted stereotactic Ommaya reservoir implantation is quick, effective, and minimally invasive. Patients have less traumatic responses, quicker recoveries, and increased qualities of life compared with those that have had traditional implantation. The technique effectively negates the inefficiencies of craniotomy and provides a novel treatment for intracranial lesions.

## Data Availability Statement

The original contributions presented in the study are included in the article/supplementary material, further inquiries can be directed to the corresponding author/s.

## Ethics Statement

The studies involving human participants were reviewed and approved by Ethics Committee of Beijing Tiantan Hospital. Written informed consent to participate in this study was provided by the participants' legal guardian/next of kin. Written informed consent was obtained from the individual(s), and minor(s)' legal guardian/next of kin, for the publication of any potentially identifiable images or data included in this article.

## Author Contributions

H-GL and D-FL performed the surgery, analyzed the data, and wrote the paper. KZ and F-GM participated in the data collection. A-CY and J-GZ designed the topic, supervised the research process, and modified the paper. All authors contributed to the article and approved the submitted version.

## Conflict of Interest

The authors declare that the research was conducted in the absence of any commercial or financial relationships that could be construed as a potential conflict of interest.
